# Lumped-Element Circuit Modeling for Composite Scaffold with Nano-Hydroxyapatite and Wangi Rice Starch

**DOI:** 10.3390/polym15020354

**Published:** 2023-01-10

**Authors:** Xiao Jian Tan, Ee Meng Cheng, Nashrul Fazli Mohd Nasir, Mohd Shukry Abdul Majid, Mohd Ridzuan Mohd Jamir, Shing Fhan Khor, Kim Yee Lee, Kok Yeow You, Che Wan Sharifah Robiah Mohamad

**Affiliations:** 1 Centre for Multimodal Signal Processing, Tunku Abdul Rahman University of Management and Technology (TAR UMT), Jalan Genting Kelang, Setapak, Kuala Lumpur 53300, Malaysia; 2 Department of Electrical and Electronics Engineering, Faculty of Engineering and Technology, Tunku Abdul Rahman University of Management and Technology (TAR UMT), Jalan Genting Kelang, Setapak, Kuala Lumpur 53300, Malaysia; 3 Sports Engineering Research Centre (SERC), Universiti Malaysia Perlis (UniMAP), Perlis 02600, Malaysia; 4 Faculty of Electronic Engineering & Technology, Universiti Malaysia Perlis (UniMAP), Perlis 02600, Malaysia; 5 Advanced Communication Engineering (ACE) Centre of Excellence, Universiti Malaysia Perlis (UniMAP), Perlis 02600, Malaysia; 6 Faculty of Mechanical Engineering & Technology, Universiti Malaysia Perlis (UniMAP), Perlis 02600, Malaysia; 7 Faculty of Electrical Engineering & Technology, Universiti Malaysia Perlis (UniMAP), Perlis 02600, Malaysia; 8 Centre of Excellence for Renewable Energy (CERE), Universiti Malaysia Perlis, Perlis 02600, Malaysia; 9 Lee Kong Chian Faculty of Engineering & Science, Tunku Abdul Rahman University, Sungai Long Campus, Jalan Sungai Long, Sungai Long City, Cheras, Kajang 43000, Selangor, Malaysia; 10 Faculty of Electrical Engineering, Universiti Teknologi Malaysia, Johor 81310, Malaysia

**Keywords:** scaffold, rice starch, hydroxyapatite, circuit, impedance

## Abstract

Mechanistic studies of the interaction of electromagnetic (EM) fields with biomaterials has motivated a growing need for accurate models to describe the EM behavior of biomaterials exposed to these fields. In this paper, biodegradable bone scaffolds were fabricated using Wangi rice starch and nano-hydroxyapatite (nHA). The effects of porosity and composition on the fabricated scaffold were discussed via electrical impedance spectroscopy analysis. The fabricated scaffold was subjected to an electromagnetic field within the X-band and Ku-band (microwave spectrum) during impedance/dielectric measurement. The impedance spectra were analyzed with lumped-element models. The impedance spectra of the scaffold can be embodied in equivalent circuit models composed of passive components of the circuit, i.e., resistors, inductors and capacitors. It represents the morphological, structural and chemical characteristics of the bone scaffold. The developed models describe the impedance characteristics of plant tissue. In this study, it was found that the ε′ and ε″ of scaffold composites exhibited up and down trends over frequencies for both X-band and Ku-band. The circuit models presented the lowest mean percentage errors of Z′ and Z″, i.e., 3.60% and 13.80%, respectively.

## 1. Introduction

Bone scaffolds are soluble materials made up of proteins for the sake of amendment of injured tissue. Bone scaffolds can be made of various materials, e.g., metals, composite materials, ceramics and polymers [1,2]. Scaffold plays a similar role as the extracellular matrix in native tissues through the aspect of their architectural, biological and mechanical features [3]. The bone scaffold enhances the migration of resident fibroblasts and adult stem cells to the injured area to increase the rate of proliferation to form new tissue. Scaffold plays the role of sending stimulating signals, e.g., growth factors, to enhance the rate of regeneration. Increments in porosity enhance osteogenesis, but this results in a reduction in mechanical properties of the scaffold, e.g., compressive strength [4]. A scaffold should have an adequate porosity level to ensure efficient nutrient and metabolite transport, without variation in the stability of the bone scaffold’s structure [5]. In addition, the biomaterial used for the production of scaffolds should be biodegradable, whereby the rate of degradation should match the production rate of new tissue matrix. Scaffolds should be biocompatible with the cellular components of the host tissue [6]. In this research, a nano-hydroxyapatite with Wangi rice starch as a biodegradable composite scaffold was chosen for the study.

Lumped-element circuit modeling is a method of using ideal electronic elements to demonstrate real physical systems. The typical elements used are resistors, capacitors, and inductors. Lumped-element modeling provides a good physical insight for a variety of systems. However, the circuit’s characteristic length is much smaller than the circuit’s operating wavelength. Lumped-element modeling can be realized at microwave frequencies [7,8,9,10]. Many past studies have used this model for various applications [11,12,13,14,15,16]. At higher frequencies, the inductive and capacitive reactance of the passive elements are significant. High frequencies lead to high loss and spurious resonance, which may result in inaccuracies of the prediction of the lumped-element circuit’s performance. Each reactance is an integral part of the component, where its effect would be considered in the design of the model. The lumped-element circuit model consists of elements which are needed for the electrical response of a physical system. The models can be constructed analytically, numerically and empirically.

The porosity levels of scaffolds can barely be identified and predicted without actual experimentation, e.g., gravimetric method, mercury intrusion method and scanning electron microscopy (SEM) method. Porosity and pore dimensions can influence the dielectric properties of the porous bone scaffold. Impedance is excellent in denoting the electrical properties of a scaffold from the perspective of porosity, and is simple to compute on the basis of the dielectric constant and loss factor. Impedance is the performance parameter in this study which can be embodied in the lumped-element circuit model to demonstrate the electrical properties of the scaffold. Lumped-element circuit modeling is advantageous, due to its simplicity and compatibility within a wide bandwidth. Hence, lumped-element circuit modeling is implemented for the behavior of the scaffold in the aspect of the porosity and composition of the bone scaffold, which is mainly composed of nano-hydroxyapatite (nHA) and rice starch. The impedance of the lumped-element model needs to be verified by comparison with measured values. The mean errors of this comparison were determined to verify the accuracy of lumped-element models. The developed lumped-element circuit models can be used to impersonate the scaffold composite in terms of the impedance that is interrelated with variations in its dielectric properties, due to porosity and structural change.

## 2. Materials and Methods

In this research, nHA and Wangi rice starch were used to fabricate the composite scaffold. Composite scaffolds made of nHA have the advantages of good biocompatibility, large specific surface area, high biological activity and stable chemical properties. Meanwhile, rice is a staple food in ASEAN countries. Wangi rice starch is ubiquitous in ASEAN countries, especially in Malaysia. In this study, commercial native cornstarch (Cs) and analytical-grade sodium chloride (NaCl) were used as matrix material and particle porogen of the composite, respectively. The hydroxyapatite nanoparticles (HA, CAS: 1306-06-5) from Sigma-Aldrich (St. Louis, MO, USA) were used as filler.

### 2.1. Sample Preparation

Table 1 tabulates the percentages of weight/volume (*w/v*%) of rice starch and nHA for preparation of the rice starch/nHA composite scaffold. In this study, Wangi rice was used. Withal, sodium chloride (NaCl) acts as a porogen to produce the biocomposite scaffold with a porous structure.

Rice starch powder was fully dissolved in distilled water. NaCl was applied to the rice starch solution to form a rice starch/NaCl mixture at a temperature of 35 °C. The mixture was stirred at 65 °C using a double boiling process. nHA powder was mixed with the rice starch/NaCl mixture to prepare the rice starch/nHA solution at a temperature of 75 °C, and the solution was stirred to ensure uniform mixing. The concentration of rice starch/nHA solution can be expressed in Equation (1) as follows:

(1) $$Concentration\;=\;\frac{mass\;of\;HA\;powder(mg)}{liter\;of\;solution(L)}$$

The rice starch/nHA composite was cast into Teflon molds, with cavity dimensions of 22.86 mm × 10.16 mm × 5 mm (WR90) and 15.8 mm × 7.9 mm × 5 mm (WR62). The cast composites in the mold were kept in an oven at 80 °C for 48 h (drying process).

After the drying process, the composites detached from the mold were soaked in 25% glutaraldehyde (GA) for 5 h (cross-linking) [17]. The immersion process in distilled water and ethanol was then continued for 24 h and 12 h, respectively. Lastly, the composites were dried in an oven at 65 °C for 48 h before measurements.

### 2.2. Measurement

Dielectric measurement was conducted using Agilent E5071C ENA vector network analyzer, in conjunction with WR90 and WR62 rectangular waveguides. Calibration was conducted using the through-reflect-line (TRL) calibration technique prior to dielectric measurement. The casted samples in the mold were placed in a sample holder based on the dimensions of WR90 and WR62. The sample with the sample holder was placed in between the pair of rectangular waveguides (WR90 and WR62) for dielectric measurement. Dimensions of the sample under testing were determined through an aperture in the WR62 and WR90 rectangular waveguides. The WR90 waveguide operates from 8.2 GHz to 12.4 GHz (X-band); meanwhile, the WR62 waveguide operates from 12.4 GHz to 18 GHz (Ku-band). The ε′ and ε″ were measured to determine complex electrical impedance.

### 2.3. Lumped-Element Modeling

In this study, the impedance analysis of the scaffold was carried out. Equations (2) and (3) express the magnitude and phase of complex impedance [18]:

(2) $$|Z|=\frac{\sqrt{\mu_{o}/\varepsilon^{\prime }}}{{\left[1+{\left(\frac{\varepsilon^{\prime \prime }}{\varepsilon^{\prime }}\right)}^{2}\right]}^{\frac{1}{4}}}$$

where

(3) $$\mu_{o}=4\pi \times 10^{-7}N/A^{2}$$

For convenience, the complex impedance of the circuit is presented in rectangular form. The real and imaginary parts of the complex impedance can be expressed as follows:

(4) $$Z*=\left|Z^{\prime }\right|-j\left|Z^{\prime \prime }\right|$$

where

(5) $$Z^{\prime }|=|Z*|\cos\theta$$

and

(6) $$|Z^{\prime \prime }|=|Z*|\sin\theta$$

Meanwhile, frequency-dependent resistance can be expressed as follows:

(7) $$R(f)=\sqrt{\frac{\pi f\mu_{0}}{\sigma }}$$

where σ is conductivity.

## 3. Results and Discussion

### 3.1. Dielectric Constant and Dielectric Loss Factor of Wangi Rice Starch/nHA Scaffold with Various Ratios at X-Band and Ku-Band

**Figure 1:**
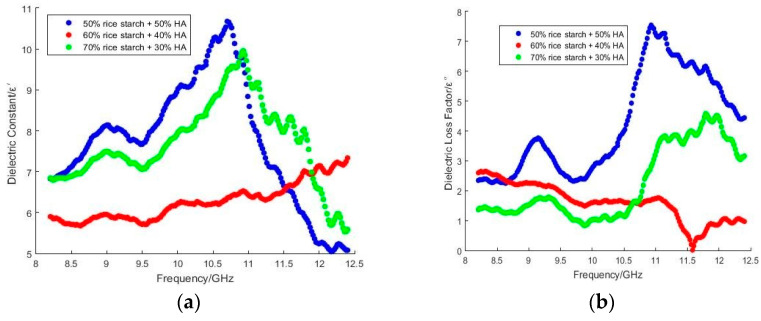
Variations in (**a**) ε′ and (**b**) ε″ over frequency (X-band) with various proportions of rice starch and nHA.

The dielectric measurements at high frequencies were conducted on the porous composites, to investigate the dielectric response due to the rapid process of molecular rearrangements and distortions in the bone scaffold composites [19,20]. The effect of the nHA and Wangi rice starch on interactions and the microstructural features of the porous bone scaffold composites were studied through dielectric analysis [21,22,23]. At high frequencies, the dipolar and interfacial polarization were described by the real part of complex permittivity (ε′). Meanwhile, the effect of energy dissipation (polarization loss and conductivity loss) were expressed by ε″ [24]. As per the Maxwell–Wagner effect, the presence of complex interfaces and dipole moments in the heterogeneous scaffold composites with rice starch and nHA were due to the dispersion and morphology of the hydroxyapatite nanoparticles and pores in the starch matrix [25,26]. The hydroxyapatite nanoparticles interacted with rice starch in the porous scaffold via storage charge and charge dissipation. This led to the polarization and the energy dissipation mechanisms of the composites [27]. The peak of ε′ in Figure 1a corresponds to a dielectric relaxation peak of ε″ in Figure 1b [28]. In Figure 1a,b, it can be observed that for ε′ and ε″, composite scaffold with compositions A and C increased from 8 GHz to 11 GHz, and decreased when the frequency was >11 GHz. This indicated the dispersion of homogeneity. It led to interfacial polarization and space charge. However, scaffold composite with composition B was exempted. This may have been due to the high homogeneity that occurred within the scaffold composite with composition B. A partially collapsed starch matrix presented in the scaffold composite. It led to reduction in polarization mechanisms. Hence, the ε′ of scaffold composite with composition B was the lowest. The SEM images of the composite scaffold with compositions A, B and C can be referred to [28].

**Figure 2:**
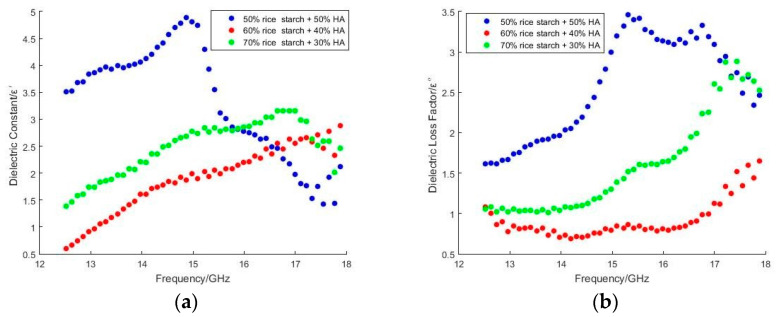
Variations in (**a**) ε′ and (**b**) ε″ over frequency (Ku-band) with various proportions of rice starch and nHA.

With dipole polarization when the frequency increased to 11 GHz, the increment in ε′ over frequency indicated that the effectiveness of alignment of polar functional groups in nHA with an operating frequency of applied field increased. The functional groups in nHA contributed to variations in ε′, especially –OH [28] and -CO_3_^2−^. These polar functional groups were involved in dipole polarization. During dipole polarization, these functional groups oscillate and vibrate by exposure to a time-varying field. Meanwhile, the non-polar functional group in nHA was -(PO_4_)^3−^. However, rice starch consists of amylose and amylopectin, which imposed an insignificant effect on the variation in ε′. The -OH groups in amylose for hydrogen bonding with polar molecules were reduced in amylose, due to their coiled nature. Meanwhile, amylopectin had low polarity due to the additional 1–6 glycosidic bonds on the branch chains. It diminished hydrogen bonding potential, and led to a low polar moment. Hence, the scaffold composite with the highest proportion of nHA (50%) was the highest, as shown in Figure 1a and Figure 2a. The non-uniform oscillation of -OH, -(PO_4_)_3_^2−^ and -CO_3_^2−^ in nHA due to polarization may have caused collisions and friction among molecules and functional groups. Subsequently, this phenomenon increased ε″. Nevertheless, ε″ declined, as shown in Figure 1b, when frequencies were >11 GHz. The stagnant polarization that led to the reduction in ε′ presented a decrement in ε″. This indicates that the process of friction and collision during oscillation or vibration was retarded. Although the variation in ε′ over the percentage of HA is an anomaly, as shown in Figure 1a, ε″ increased over the percentage of HA within frequencies < 11 GHz. When frequencies were >11 GHz, ε′ of scaffold composite with 60% rice starch + 40% HA at X-band exceeded those of 50% rice starch + 50% HA and 70% rice starch + 30% HA. Meanwhile, ε′ of scaffold composite with 60% rice starch + 40% HA at X-band was lower than those of 50% rice starch + 50% HA and 70% rice starch + 30% HA. This inconsistency may be attributed to the transition of the polarization mechanism.

The ε′ of scaffold composite with 50% rice starch + 50% HA was the highest at 11 GHz. At 11 GHz, resonance occurred due to the consistency between the applied field and relaxation frequency of the scaffold composite with the highest proportion of HA. It had the most effective absorption capacity and polarization by exposure to the oscillating field.

The ε′ at Ku-band, as shown in Figure 2a, was relatively lower than that for the X-band. High frequencies at Ku-band led to a high discrepancy between the operating frequency of the applied field and the relaxation frequency of the bone scaffold. As a result, the polarization mechanism was stagnant. This response caused a low ε″ at Ku-band, as shown in Figure 2b. The vertex of ε″ can be noticed over frequency in both Ku-band and X-band. It implies the transition of polarization mechanisms.

Likewise, a similar anomaly can be noticed in Figure 2a,b. The ε′ and ε″ of the scaffold composite with 60% rice starch + 40% HA at Ku-band were lower than those for 50% rice starch + 50% HA and 70% rice starch + 30% HA. The strength of the bone scaffold relies on either agglomeration HA or recrystallization of amylose and amylopectin (starch). Rapid recrystallization of amylose molecules and slow recrystallization of amylopectin molecules occurred during the retrogradation process of starch. Meanwhile, the agglomeration of HA occurred due to electrostatic forces between HA in the nanoscale. These phenomena helped strengthen the structure of scaffold composite. However, the strength of the composite was largely subject to applying the proper ratio of rice starch to HA. It can be speculated that a scaffold composite with 60% rice starch + 40% HA has a weak structural wall that is fragile and tends to collapse. This phenomenon causes a switch of the main polarization mechanism, i.e., space charge and interfacial polarization of dipole polarization due to one polar functional group to the other. As a result, the response of ε′ and ε″, as shown in Figure 1 and Figure 2, is an anomaly.

### 3.2. Lumped-Element Circuit Model

**Figure 3:**
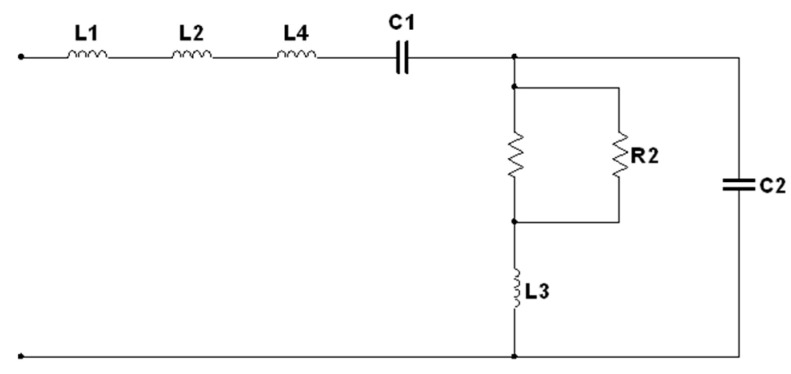
Circuit model at X-band.

**Figure 4:**
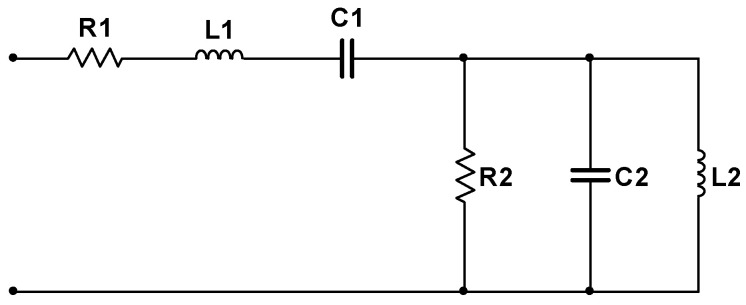
Circuit model at Ku-band.

The lumped-element circuit models in Figure 3 and Figure 4 were designed to simulate the electrical properties of the scaffold composite with Wangi rice starch and nHA at X- band and Ku-band, respectively. The specifications of the circuit models at X-band and Ku-band are tabulated in Table 2 and Table 3.

**Figure 5:**
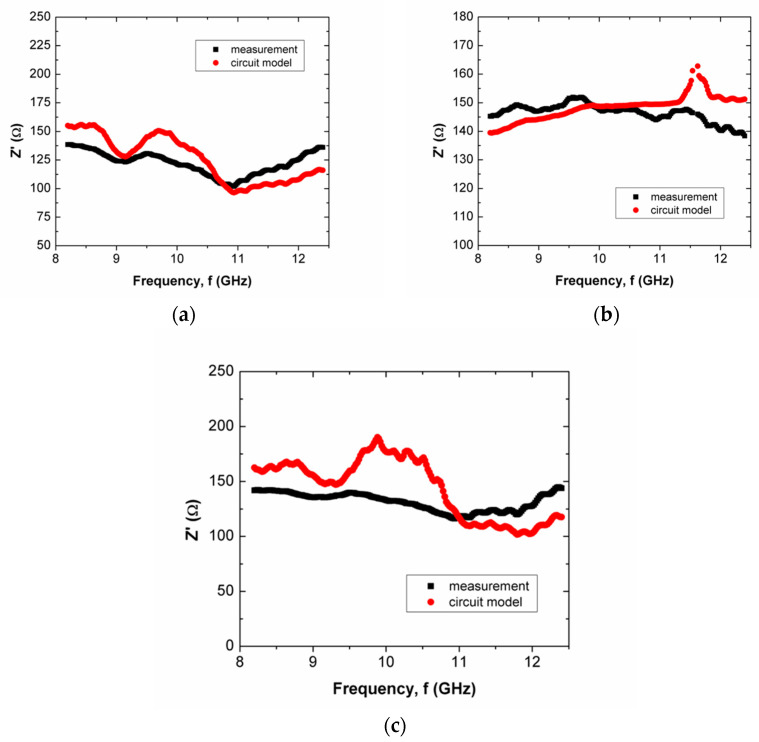
Comparison between measured and simulated Z′ (real part of impedance) of scaffold composites with (**a**) 50% Wangi rice starch and 50% nHA, (**b**) 60% Wangi rice starch and 40% nHA and (**c**) 70% Wangi rice starch and 30% nHA, at X-band.

**Figure 6:**
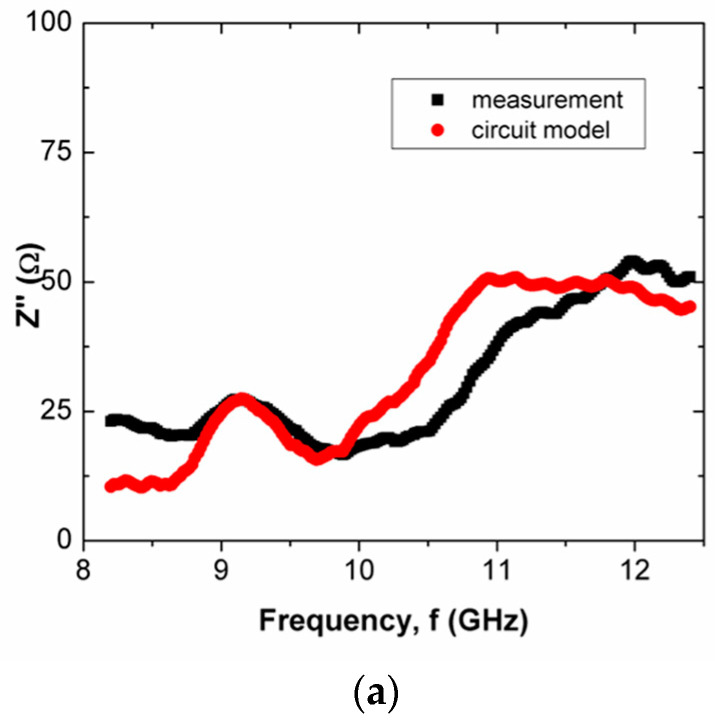

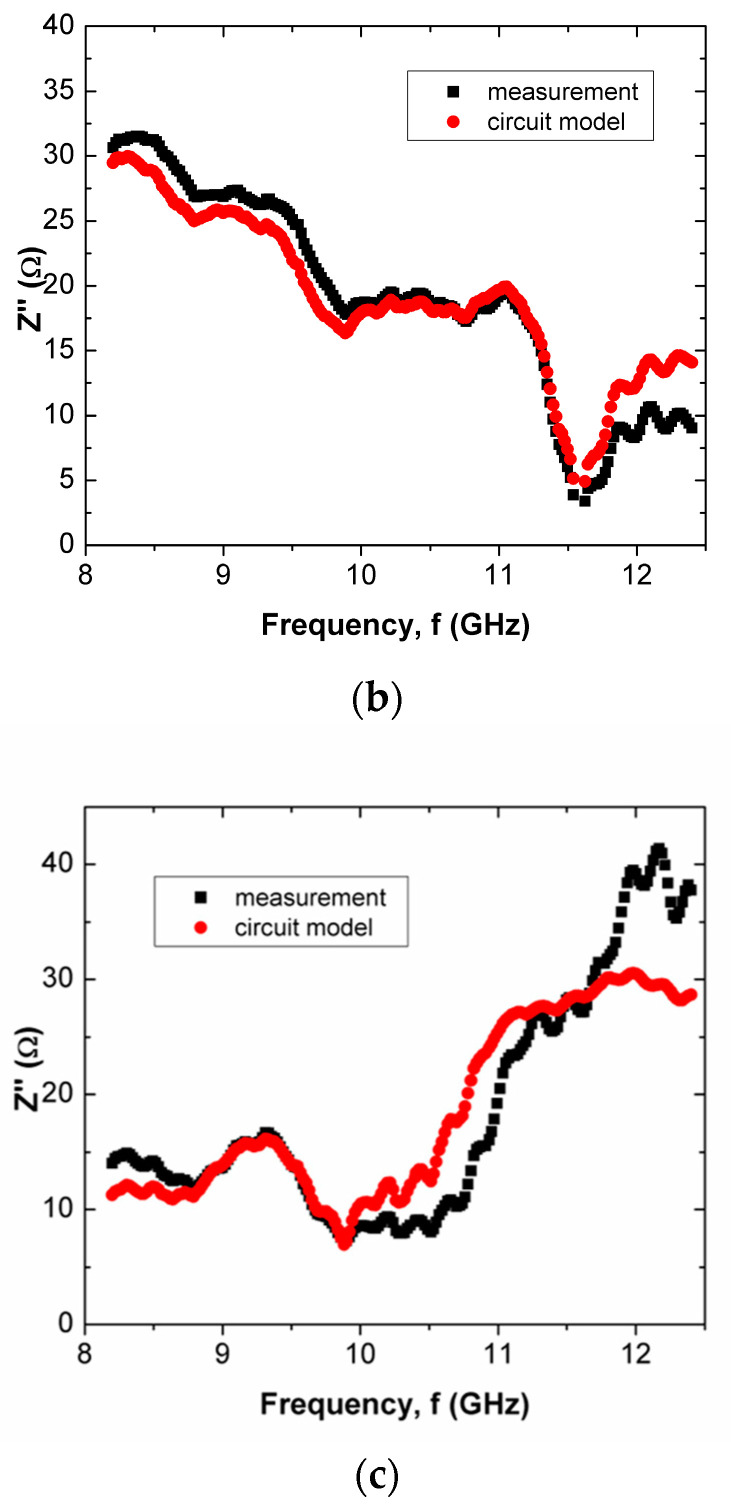
Comparison between measured and simulated Z″ (imaginary part of impedance) of scaffold composites with (**a**) 50% Wangi rice starch and 50% nHA, (**b**) 60% Wangi rice starch and 40% nHA and (**c**) 70% Wangi rice starch and 30% nHA, at X-band.

**Figure 7:**
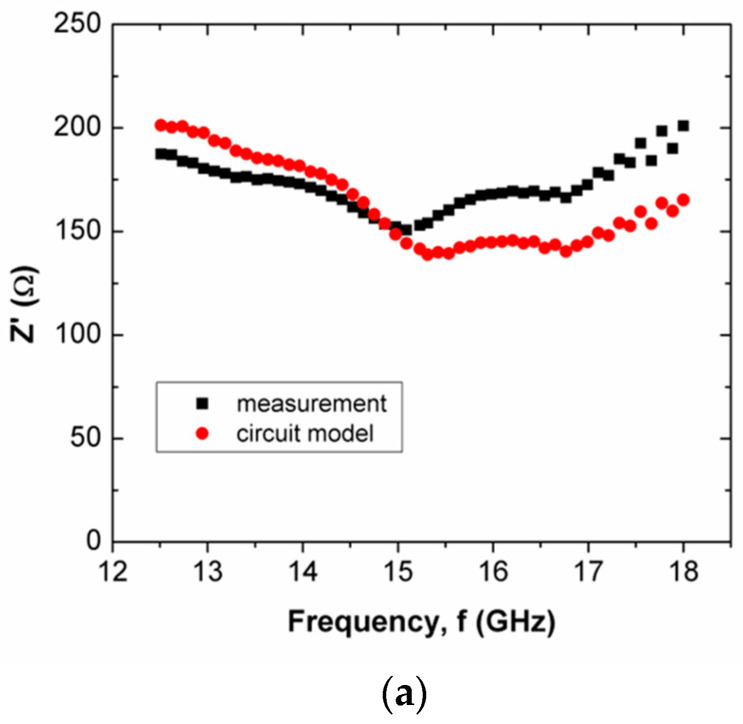

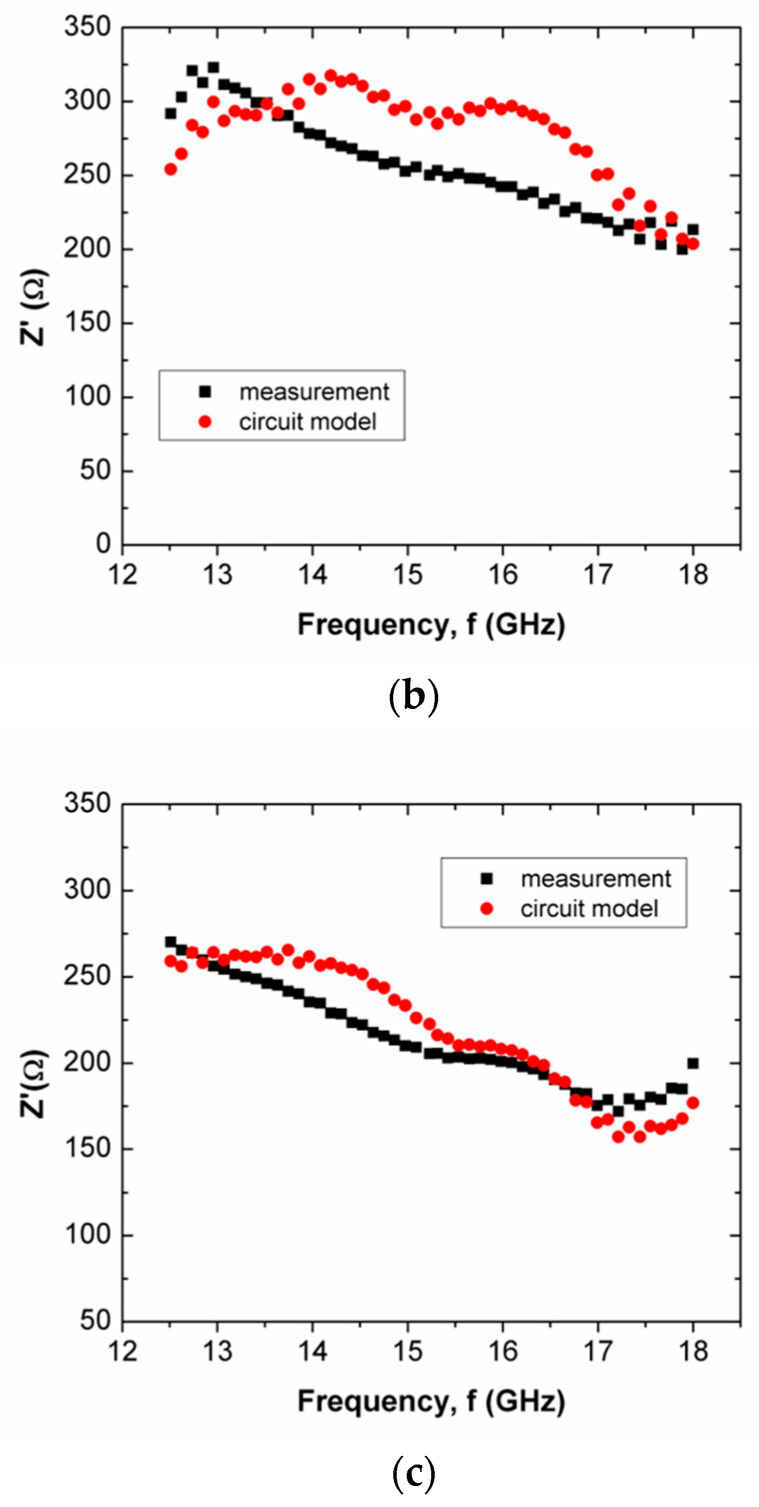
Comparison between measured and simulated Z′ of scaffold composites with (**a**) composition A, (**b**) composition B and (**c**) composition C, at Ku-band.

**Figure 8:**
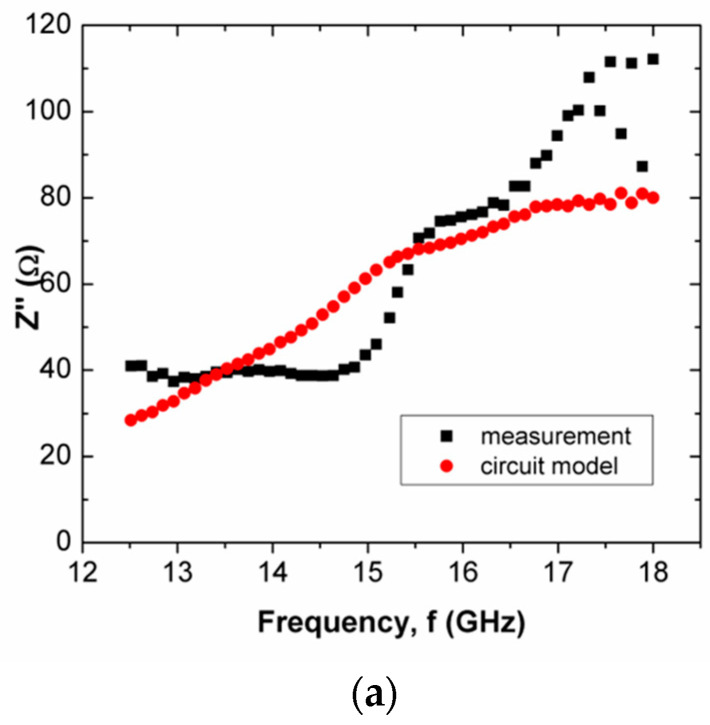

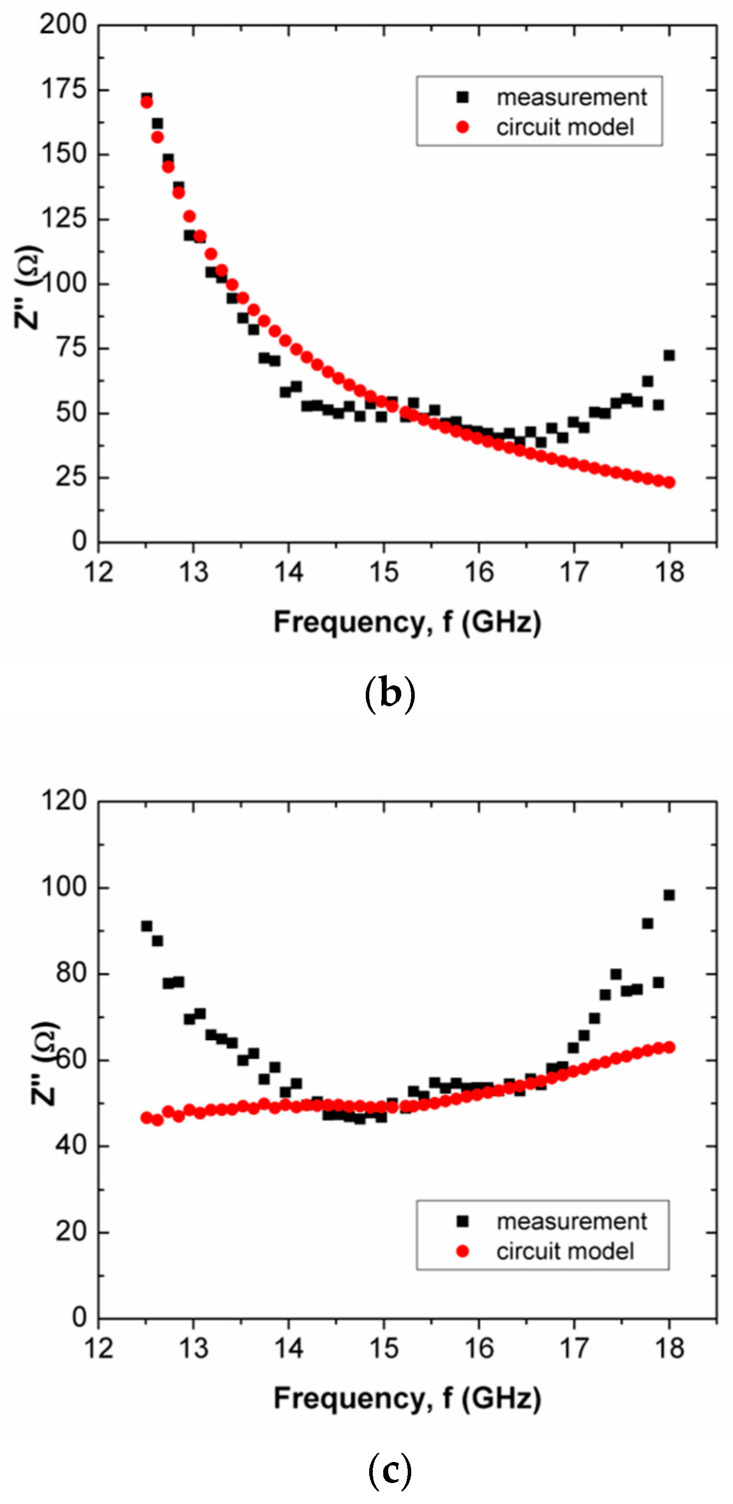
Comparison between measured and simulated Z″ of scaffold composites with (**a**) composition A, (**b**) composition B and (**c**) composition C, at Ku-band.

The Z′ and Z″ of scaffold composites over frequency are shown in Figure 5, Figure 6, Figure 7 and Figure 8 for scaffold composites with 50% rice starch + 50% nHA (composition A), 60% rice starch + 40% nHA (composition B) and 70% rice starch + 30% nHA (composition C) at X-band and Ku-band. Z′ and Z″ imply the DC and AC characteristics of the circuit model. Typically, a resistor has a resistance that is frequency-independent. However, resistance becomes frequency-dependent (=R(f)), as expressed in Equation (7), at high frequencies. The variation in Z′ over frequency is insignificant. It is similar to the resistance of a resistor in the DC response. The narrow frequency range at either X-band or Ku-band leads to insignificant variation. It is mainly due to the time-invariant average power of the applied field. In other words, the variation in Z′ is attributed to the DC effect of time-variance or the electromagnetic field. The presence of frequency-independent resistor R2, as shown in Figure 3, reduces the impact of R(f) towards Z′ and Z″. It can be noticed that R2 plays an effect in reducing the total resistance of the circuit for the scaffold composite with 70% rice starch + 30% nHA. This may be due to the retrogradation of the 70% rice starch-prepared scaffold composite with a short oscillation distance (short diameter of the pore).

When the operating frequency of the applied field corresponds with the relaxation frequency of the scaffold composite, the charge in the pore can oscillate within the pore that is presented in the scaffold composite, and this leads to the presence of space charge. This charge is responsible for interfacial polarization. More oscillation occurs simultaneously in high porosities of scaffold composites than in low porosities. Apparently, the scaffold composite with 60% rice starch + 40% nHA (composition B) exhibited lower porosity than those of 50% rice starch + 50% nHA (composition A) and 70% rice starch + 30% nHA (composition C). The interfacial polarization facilitated charge migration and oscillation within the body, as well as within the pore. The rate of change in electric potential difference (voltage) due to the presence of space charge on grain boundary increased, and this increased current. Consequently, the increment in current implied that resistance decreased. Hence, it is explicable that a low resistance value of R2 is applied to mitigate the impact of R(f) on Z′. On the other hand, it can be seen that the mean percentage error of Z′ of the scaffold composite with composition C was the highest (i.e., 18.31%), as tabulated in Table 4. Generally, the Z′ of the circuit model has better agreement with the measured Z′ for X-band and Ku-band, as shown in Figure 5a and Figure 7a, respectively. This can be noticed through Table 4 and Table 5, where the mean percentage errors of Z′ are lower than Z″ for X-band and Ku-band. These may be attributed to the lower variabilities in Z′ than Z″, where the AC resistance R(f) and DC resistance R have a significant and direct effect on Z′. Meanwhile, Z″ is a function of frequency, inductance and capacitance, depending on the circuit network.

The mean percentage error of Z″ of composition C was considerably high, i.e., 19.80%. This proportion provided the strongest inner wall structure. Hence, it had substantial well-established and distributed pores. This increased the complexity of the internal structure of the scaffold composite, which led to a severe discrepancy of Z′ and Z″ between the circuit model and measurement, that was reflected by the highest mean percentage error.

When the scaffold composite had low porosity, charge migration in the thick inner wall was attributed to oscillation during polarization. At high frequencies, charge migration may have been stagnant due to operating frequencies that exceeded relaxation frequencies. Hence, the resistance of composition B at Ku-band had a higher resistance than that at X-band.

Z″ is frequency dependent. It can be capacitive or inductive. The Z″s of scaffold composites composition A and composition C were inductive, where Z″ increased over frequency. Meanwhile, scaffold composite composition B exhibited a capacitive characteristic, where Z″ decreased when frequency increased. Both proportions of rice starch to nHA indicated an increment in inductive effect that led to a decrement in capacitive effect. This may have been due to the oscillation of charge within a substantial number of pores. At X-band and Ku-band (high frequency, ~10^9^ Hz), the variation in charge on the grain boundary in the pore induced a magnetic field that accounted for inductive behavior. It caused a loss of energy in polarization, which accounted for the capacitive effect. In the high-frequency range, the rate of charge change in the pores was high. It generated a considerably large current. This implies the presence of significant inductive characteristics at X-band and Ku-band. It can be clearly seen in Figure 6a,c and Figure 8a,c. However, scaffold composite with composition B (60% rice starch + 40% nHA) had fewer pores to act as a platform for polarization. Hence, the effect of dielectric polarization on the Z″ was less significant.

It can be seen that composition C at Ku-band exhibited a lower mean percentage error than X-band for Z′ (6.78%) and Z″ (13.80%). This was noticed through a comparison of the mean percentage errors between X-band (Table 4) and Ku-band (Table 5). The designed circuit model at Ku-band was less complex than that for X-band. Z″ over frequencies <15.5 GHz and >15.5 GHz, as shown in Figure 8c, exhibited negative and positive gradients, respectively.

The major switch of gradient from negative to positive within this narrow frequency range (Ku-band) cannot be represented by a single circuit model. It can be justified by the low coefficient of determination (R^2^) of Z″ for composition Cat Ku-band (R^2^ = 0.19). As a result, the same circuit model, as shown in Figure 4 for frequencies <15.5 GHz and >15.5 GHz, can still be applied. However, new component values for frequencies <15.5 GHz and >15.5 GHz are tabulated in Table 5. Table 6 shows the new component values of the circuit model (composition C) in Figure 4 for frequency ranges <15.5 GHz and >15.5 GHz.

**Figure 9:**
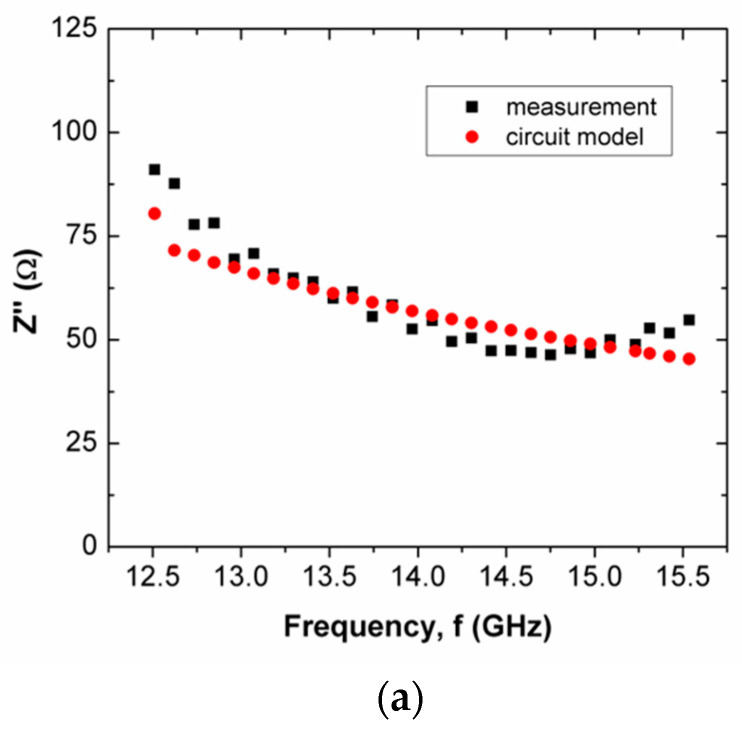

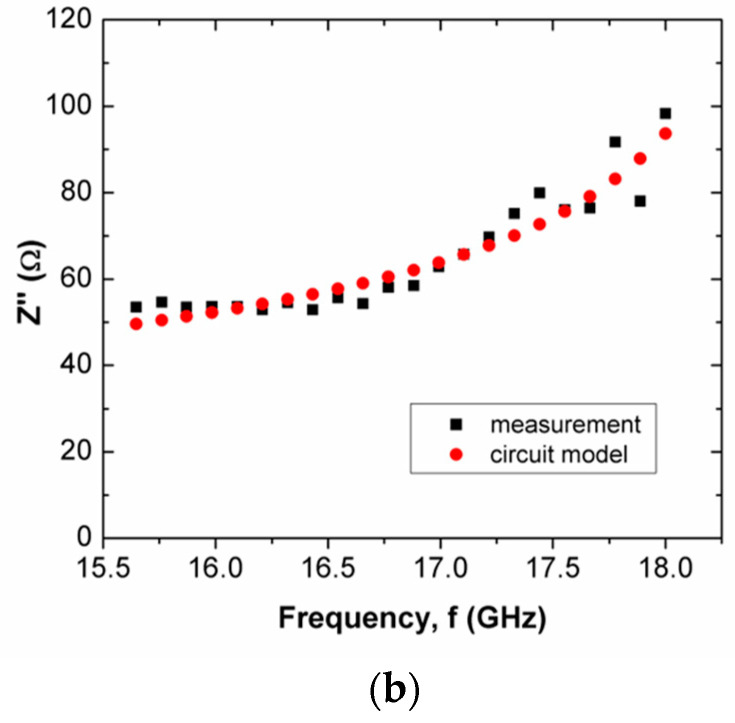
Measured and simulated Z″s of the scaffold composite with composition C for frequencies (**a**) <15.5 GHz and (**b**) >15.5 GHz, at Ku-band.

When the frequency was <15.5 GHz, the capacitive effect reduced as Z″ decreased over frequencies where the increment of inductive effect mitigated the capacitive effect, as shown in Figure 9a. It can be seen in Table 5 that the value of L2 is smaller than C2 in the parallel network. The impact of L2 in this parallel network had a more significant effect on the Z″ than that of C2. The storage of electrical energy during polarization decreased due to the delay of polarization mechanisms when the frequency increased gradually to 15.5 GHz. This delay may be ascribed to the immobilization of the hydroxyl group (−OH), due to the recrystallization of amylose and amylopectin. The decrement ceased at 15.5 GHz, and began increasing at frequencies above 15.5 GHz. The Z″ increased when frequencies were >15.5 GHz, as shown in Figure 9b. The polarization mechanism involved the oscillation of charges in the pore with the applied field. The recrystallization of amylose and amylopectin with 70% rice starch in the scaffold composite prepared sufficient pores as a platform for space charge or interfacial polarization. The charge could oscillate synchronously with the applied field polarization when frequencies were >15.5 GHz. This phenomenon occurred in the scaffold composite with composition C (70% rice starch + 30% nHA) within the high-frequency range (Ku-band). In regards to the circuit, L1 and L2 exerted a significant impact on Z″, causing Z″ to increase with frequency. This gradient switch was not observable at X-band, since the frequency range of X-band is lower than that of Ku-band. The Z″ of the circuit model, as shown in Figure 9a,b, demonstrated good agreement with measurement. This was observed through the mean percentage error of Z″. The mean percentage error and R2 of Z″ for frequencies <15.5 GHz were 7.35% and 0.85, respectively. For frequencies >15.5 GHz, the mean percentage error and R2 of Z″ were 4.90% and 0.90, respectively.

## 4. Conclusions

This study discussed composite scaffolds with nano-hydroxyapatite and Wangi rice starches using lumped-element circuit models. Scaffold composites with Wangi rice starch and nHA were fabricated. The porosity level of this biodegradable composite was identified through impedance analyses in electromagnetic fields. In this research, Z′ and Z″ of these designed circuit models were found to be in good agreement with the measurement where the mean percentage error was <25%. However, the mean percentage error of composition C for Ku-band was higher than that for X-band. It was surmised that the weak structural wall was sensitive to frequency. A minor difference was noticed between X-band and Ku-band in terms of the designed circuits. However, distinctive component values of both circuits were noticed due to different frequency ranges (X-band and Ku-band). The developed model is useful to determine the electromagnetic behavior of the biomaterial, which can be associated with morphological, structural and chemical characteristics. A similar model can be formed and used for other composite biomaterials of interest.

## Figures and Tables

**Table 1:** Weight/volume percentages (*w/v*%) of nHA and Wangi rice starch used to fabricate the scaffold.

Composition	nHA (*w/v*%)	Rice Starch Concentration (*w/v*%)
A	50	50
B	40	60
C	30	70

A is 50% rice starch + 50% nHA; B is 60% rice starch + 40% nHA; C is 70% rice starch + 30% nHA.

**Table 2:** Component values of the lumped-element circuit (Figure 3) for scaffold composites of Wangi rice starch and nHA at X-band.

Composition	Component Values in the Circuit (Figure 3)							
	L1 (H)	L2 (H)	L3 (H)	L4 (H)	C1 (F)	C2 (F)	R1 (Ω)	R2 (Ω)
A	7.24 × 10^−10^	3.27 × 10^−11^	3.16 × 10^−10^	-	1.00 × 10^−9^	3.60 × 10^−14^	R(f)	≥8000
B	3.30 × 10^−11^	3.00 × 10^−12^	1.00 × 10^−9^	1.70 × 10^−10^	1.00 × 10^−9^	7.00 × 10^−14^	R(f)	400
C	5.00 × 10^−10^	3.00 × 10^−11^	1.00 × 10^−12^	-	3.44 × 10^−8^	1.14 × 10^−14^	R(f)	604

**Table 3:** Component values of the lumped-element circuit (Figure 4) for scaffold composites of Wangi rice starch and nHA at Ku-band.

Composition	Component Values in the Circuit (Figure 4)					
	L1 (H)	L2 (H)	C1 (F)	C2 (F)	R1 (Ω)	R2 (Ω)
A	1.20 × 10^−9^	9.00 × 10^−8^	3.16 × 10^−13^	1.00 × 10^−14^	0	R(f)
B	1.69 × 10^−10^	6.00 × 10^−10^	<1.00 × 10^−6^	3.39 × 10^−13^	R(f)	>8000
C	1.00 × 10^−9^	1.00 × 10^−8^	2.00 × 10^−13^	1.00 × 10^−14^	0	R(f)

**Table 4:** Components values of the lumped-element circuit (Figure 3) for scaffold composites of Wangi rice starch and nHA at X-band.

Composition	Mean Percentage Error (%)	
	Z′	Z″
A	11.09	23.90
B	3.60	13.57
C	18.31	19.80

**Table 5:** Mean percentage errors for comparison between measured and simulated Z′ and Z″ for scaffold composites at Ku-band.

Composition	Mean Percentage Error (%)	
	Z′	Z″
A	10.23	17.21
B	12.86	23.30
C	6.78	13.80

**Table 6:** New component values of the circuit model (composition C) in Figure 4 for frequency ranges <15.5 GHz and >15.5 GHz.

Composition	Component Values in Circuit					
	L1 (H)	L2 (H)	C1 (F)	C2 (F)	R1 (Ω)	R2 (Ω)
<15.5 GHz	0	1.00 × 10^−9^	<1.00 × 10^−9^	3.20 × 10^−13^	R(f)	>8000
>15.5 GHz	3.50 × 10^−10^	5.00 × 10^−11^	<1.00 × 10^−9^	1.40 × 10^−12^	R(f)	>8000

## Data Availability

The data presented in this study are available on request from the corresponding author. The data are not publicly available due to their confidentiality.
